# Assessing the Europe 2020 Strategy Implementation Using Interval Entropy and Cluster Analysis for Interrelation between Two Groups of Headline Indicators

**DOI:** 10.3390/e23030345

**Published:** 2021-03-15

**Authors:** Natalja Kosareva, Aleksandras Krylovas

**Affiliations:** Department of Mathematical Modelling, Vilnius Gediminas Technical University, Saulėtekio al. 11, 10223 Vilnius, Lithuania; aleksandras.krylovas@vilniustech.lt

**Keywords:** Europe 2020 strategy, EU-28 countries, smart, sustainable and inclusive growth, headline indicators, WEBIRA, interval entropy, cluster analysis

## Abstract

The research analyzes the progress of Member States in the implementation of Europe 2020 strategy targets and goals in 2016–2018. Multiple criteria decision-making approaches applied for this task. The set of headline indicators was divided into two logically explained groups. Interval entropy is proposed as an effective tool to make prioritization of headline indicators in separate groups. The sensitivity of the interval entropy is its advantage over classical entropy. Indicator weights were calculated by applying the WEBIRA (weight-balancing indicator ranks accordance) method. The WEBIRA method allows the best harmonization of ranking results according to different criteria groups—this is its advantage over other multiple-criteria methods. Final assessing and ranking of the 28 European Union countries (EU-28) was implemented through the α-cut approach. A k-means clustering procedure was applied to the EU-28 countries by summarizing the ranking results in 2016–2018. Investigation revealed the countries–leaders and countries–outsiders of the Europe 2020 strategy implementation process. It turned out that Sweden, Finland, Denmark, and Austria during the three-year period were the countries that exhibited the greatest progress according to two headline indicator groups’ interrelation. Cluster analysis results are mainly consistent with the EU-28 countries’ categorizations set by other authors.

## 1. Introduction

Europe 2020 is the EU’s agenda for smart, sustainable, and inclusive growth. The goals are to deliver high levels of employment, productivity, and social cohesion in the Member States while reducing the impact on the natural environment [1]. Ambitious targets in the areas of employment, research and development (R&D), climate change, energy, education, and poverty reduction, to be reached by 2020, were set by the EU. Nine headline indicators show how fast each Member State is reaching overall targets and how much more effort is needed to achieve these targets.

There have been a number of publications on Europe 2020 strategy in the last few years. In many publications, the authors suggest comparable composite indicators to assess the performance of the Member States with respect to implementation of the goals described by the Europe 2020 strategy—see, for example, [2,3,4,5,6,7,8,9,10,11].

Some of the publications pay special attention to the results of individual countries or groups of countries in the implementation of the Europe 2020 strategy. Thus, in [11], diversity between new Member States that joined the European Union in 2004 and 2007 (EU-10) and old European Union Members (EU-15) with special concentration on the situation in the Visegrad Group as the biggest economies of the EU-10, has been analyzed. The aim of [10] was to evaluate and compare the level of regional development in the selected EU countries (Czech Republic, Poland, Slovakia). The emphasis on the southern countries of the European Union is made in [12], on the Baltic states in [13], and on the situation in Poland and Slovakia in [14]. The disparities between the highly developed western countries and south European countries in the level of implementation of the strategy are revealed in [9]. Article [15] focused on levels of competitiveness in EU countries and especially in Slovakia. Heterogeneity gaps and pure performance differences between old and new European members are analyzed in [5].

Some articles do not cover the trends in progress of all headline indicators, but only certain optional indicators have been selected. The authors were interested in these areas of Europe 2020 strategy development: the performance of education systems [3,12], poverty and social exclusion [16], research and development management [14], unemployment [17], resource efficiency [8], issues of the labor market, income disparities and poverty [18], and greenhouse gas emissions [13].

Research methods most often used in articles are as follows: statistical methods [2,9,14,15,17,19,20], multiple-criteria decision making [4,8,10,11], the DEA (data envelopment analysis) method [3,18], and fuzzy goal programming [7].

Multiple-criteria decision-making approach is a convenient methodology in solving problems like this, where alternatives need to be assessed against certain, often conflicting, criteria (indicators). In the study by Fedajev et al. [4], the progress made in achieving the Europe 2020 goals has been evaluated using the MULTIMOORA (the full multiplicative form of multiobjective optimization by a ratio analysis) method and the Shannon Entropy Index. The study period covers 2016. The resource-efficiency capacity index (RECI) proposed in [8], measures the progress of the member states in these three dimensions: (i) transforming the economy, (ii) improving buildings, and (iii) ensuring efficient mobility. The VIKOR (visekriterijumska optimizacija i kompromisno resenje) method elaborated in [10] was used to evaluate and compare the level of regional development in the selected EU countries in the period 2010–2013.

As the evaluation of the Europe 2020 strategy implementation is a multidimensional and multilayered task, the cross-sections of individual evaluation criteria, countries or groups of countries, over different periods, can be assessed. To get a complete picture, the problem needs to be considered from different perspectives.

The intention of the authors of this article is to analyze the data on the Europe 2020 strategy priorities of smart, sustainable, and inclusive growth, divide the set of headline indicators into two logically explained groups (subsets) and rank European countries according to interrelations between two groups of indicators. Europe 2020 headline indicators have been chosen to monitor countries’ progress towards the strategy’s targets. The data on the Europe 2020 headline indicators has been gathered from the Eurostat database (see [21]). Due to the latest data availability, the analysis is based on the time period for the years 2016 to 2018, for which the data for all nine headline indicators are published.

Interval entropy was introduced as an effective tool to make prioritization of headline indicators in separate groups. Interval entropy responds adequately to small changes of initial data. The sensitivity of this method is its advantage compared with classical entropy.

In order to evaluate the weights of headline indicators, the multiple-criteria decision-making approach WEBIRA (weight-balancing indicator ranks accordance) was used. The WEBIRA method requires solving the optimization problem. In the sense of this task, it allows the best harmonization of different groups criteria and this is its advantage over other methods. Note that in this article, the goal function of the optimization task is constructed on the basis of calculated ranks. Therefore, the rankings obtained from the criteria of the different groups should be best matched to each other, compared to other methods that do not formulate such goals.

Final assessing and ranking of the EU-28 countries was implemented through the α-cut approach, which assigns ranks to countries according to the interrelation between two groups of indicators. The results of k-means cluster analysis are generally consistent with results obtained in the research of other authors.

The rest of the article is organized as follows. Section 2 presents an overview of Europe 2020 strategy targets and headline indicators. Section 3 includes decision-making matrices –values of indicators for 2016–2018, the concept of interval entropy, and the WEBIRA method for calculation criteria weights. In Section 4, the main results of the paper are provided. Section 5 is devoted to discussion on the problems considered in the article, while Section 6 includes conclusions and recommendations for future work.

## 2. Overview of Europe 2020 Strategy Targets and Headline Indicators

There are three mutually reinforcing priorities of smart, sustainable, and inclusive growth defined in the Europe 2020 strategy. The strategy’s targets and goals related to these priorities are as follows:

- Increasing combined public and private investment in research and development (R&D) to 3% of GDP (gross domestic product);
- Reducing school drop-out rates to less than 10%;
- Increasing the share of the population aged 30–34 having completed tertiary education to at least 40%;
- Reducing greenhouse gas emissions by at least 20% compared to 1990 levels;
- Increasing the share of renewable energy in final energy consumption to 20%;
- Moving towards a 20% increase in energy efficiency;
- Increasing the employment rate of the population aged 20–64 to at least 75%;
- Lifting at least 20 million people out of the risk of poverty and social exclusion.

Every EU member state declares its own national targets that generally do not coincide with Europe 2020 strategy targets. National targets reflect each country’s economy, energy consumption, innovation, and education levels and are more or less ambitious, but altogether they have to ensure implementation of the Europe 2020 strategy. Europe 2020 headline indicators are divided into five groups: employment; research and development; climate change and energy; education; poverty and social exclusion. Headline indicators C_1_–C_9_, their values for EU-28 countries in 2016–2018, and targets to be attained by 2020, are presented in Table 1.

**Figure 1:**
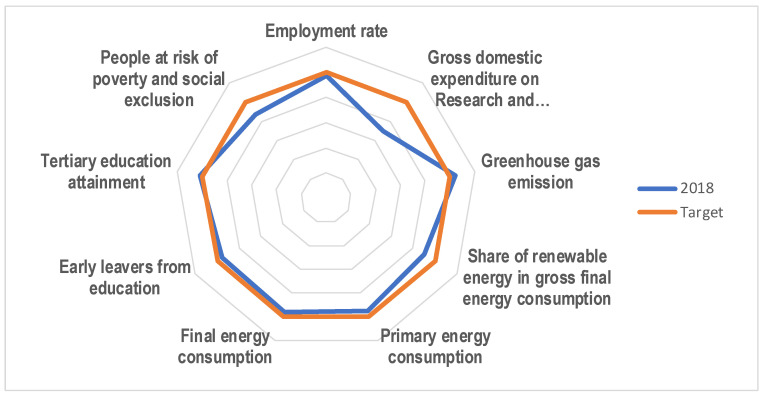
Radar chart for EU-28 countries’ Europe 2020 headline indicator values in 2018 and their targets.

The chart on Figure 1 was drawn using the data given in Table 1. The radar chart shows how far EU-28 countries are from corresponding targets as a percentage of the targets reached. Targets are denoted as red line, and the EU-28 situation in 2018 as blue line. The distance between the blue line and the red line for each indicator shows how far the EU-28 was from the target in 2018. If 2018 data points (blue line) are outside the red line, it means that this target has been exceeded, while data points inside show that progress wasn’t sufficient. Let us notice that targets were exceeded for greenhouse gas emission and tertiary education attainment. Other indicators were not sufficiently improved in 2018. The most significant retardation is noticeable for gross domestic expenditure on research and development (percentage of GDP on R&D), people at risk of poverty and social exclusion and share of renewable energy in gross final energy consumption.

It is worth mentioning that the biggest differences between countries’ targets are for gross domestic expenditure on research and development (minimum 0.5% for Cyprus, maximum 3.76% for Austria) and share of renewable energy in gross final energy consumption (minimum 10% for Malta, maximum 46% for Sweden).

## 3. Materials and Methods

### 3.1. Description of the Europe 2020 Headline Indicators

In the calculations below, units of measure for some indicators were changed in order to make data from different countries comparable. Primary energy consumption and final energy consumption (measured in million tonnes of oil equivalent (TOE)) were recalculated to million TOE per capita. Data for people at risk of poverty and social exclusion (measured in millions of people) were recalculated to percent of population.

C_1_—Employment rate. Unit of measure: percentage of population aged 20 to 64. Employed persons are defined as persons who during the reference week were working at least one hour for pay, or were not working but had jobs from which they were temporarily absent. Paid employment contributes to economic performance, quality of life, and social inclusion. It is a base of socioeconomic development and well-being.

C_2_—Gross domestic expenditure on research and development. Unit of measure: percentage of gross domestic product. “Research and experimental development (R&D) comprise creative work undertaken on a systematic basis in order to increase the stock of knowledge, including knowledge of man, culture, and society, and the use of this stock of knowledge to devise new applications [22].”

C_3_—GHG (greenhouse gas) emissions. Unit of measure: index 1990 = 100. The indicator measures total national emissions of greenhouse gases, including carbon dioxide (CO_2_), methane (CH_4_), nitrous oxide (N_2_O), and the so-called F-gases (hydrofluorocarbons, perfluorocarbons, nitrogen trifluoride (NF_3_), and sulfur hexafluoride (SF_6_)) from all sectors of the GHG emission inventories. Using each gas’s individual global warming potential (GWP), they are being integrated into a single indicator expressed in units of CO_2_ equivalents.

C_4_—Share of renewable energy in gross final energy consumption. Unit of measure: percent. The gross final energy consumption is the energy used by end consumers, plus grid losses and self-consumption of power plants.

C_5_—Primary energy consumption. Unit of measure: million tonnes of oil equivalent (TOE) per capita. The indicator measures the energy consumption by end users, such as industry, transport, households, services, and agriculture, plus the energy consumption of the energy sector itself for production and transformation of energies, losses occurring during the transformation of energies, and the transmission and distribution losses of energy.

C_6_—Final energy consumption. Unit of measure: million tonnes of oil equivalent (TOE) per capita. Covers only the energy consumed by end users, such as industry, transport, households, services and agriculture; it excludes energy consumption of the energy sector itself and losses occurring during transformation and distribution of energy.

C_7_—Early leavers from education and training. Unit of measure: percent of population aged 18 to 24. The share of the population aged 18 to 24 with, at most, lower-secondary education, who were not involved in any education or training during the four weeks preceding the survey. People with a low level of education may not only face greater difficulties in the labor market but also have a higher risk of poverty and social exclusion.

C_8_—Tertiary education attainment. Unit of measure: percent of population aged 30 to 34. The share of the population aged 30–34 who have successfully completed tertiary studies (e.g., university, higher technical institution, etc.). High levels of education boost productivity, innovation, and competitiveness.

C_9_—People at risk of poverty and social exclusion. Unit of measure: percent of population. This indicator corresponds to the persons who are: at risk of poverty after social transfers, severely materially deprived, or living in households with very low work intensity.

In Table 2, Table 3 and Table 4, the initial decision-making matrices data—Europe 2020 strategy headline indicator values in 2016–2018—are presented. The main results of the article are provided in Section 3.

Some indicators in our research must be maximized (C_1_, C_2_, C_4_, C_8_) and others (C_3_, C_5_, C_6_, C_7_, C_9_)—minimized. Before applying the WEBIRA method, the set of indicators was divided into two groups. Indicators C_3_, C_4_, C_5_, C_6_ which describe climate change and energy, i.e., the amount of energy consumed and its structure as well as the exhaust gas emissions, were assigned to the first group X. The remaining indicators C_1_, C_2_, C_7_, C_8_, C_9_ describe the social-economic situation of countries and the situation in education. They were assigned to the second group Y.

The next step is data normalization and criteria priority setting in each group. To compare the results with the EU-28 countries ranking results obtained in [4], the same vector normalization formulas for criteria to be maximized (1) and for criteria to be minimized (2) were applied to the initial decision-making matrix data x_ij_:

(1) $${\tilde{x}}_{\mathrm{ij}}=\frac{x_{\mathrm{ij}}}{\sqrt{\sum_{j=1}^{m}x_{\mathrm{ij}}^{2}}}\;,\;i=1,2,\ldots ,n,\;j=1,2,\ldots ,m,$$

(2) $${\tilde{x}}_{\mathrm{ij}}=1-\frac{x_{\mathrm{ij}}}{\sqrt{\sum_{j=1}^{m}x_{\mathrm{ij}}^{2}}}\;,\;i=1,2,\ldots ,n,\;j=1,2,\ldots ,m.\;$$

Here n = 9 is the number of headline indicators and m = 28 is the number of alternatives (countries). In Table 5, normalized data matrix for 2016 is presented. Normalized data for 2017–2018 are calculated similarly.

Hereinafter, interval entropy is applied to identify the priorities of indicators in each group. Interval entropy indicates strategic priorities where the biggest differences amongst the European countries have been identified.

### 3.2. The Concept of Interval Entropy

The entropy concept was first introduced in thermodynamics, by the German physicist Rudolf Clausius in 1850 [23], as the measure of a system’s thermal energy per unit temperature that is unavailable for doing useful work. The concept of entropy provides deep insight into the direction of spontaneous change for many everyday phenomena.

In information theory, the entropy of a random variable is the average level of information or uncertainty in the variable’s possible outcomes. The concept of information entropy was introduced by Claude Shannon in 1948 [24].

In 1949 Edward Guggenheim [25], defined entropy as a measure of energy dispersal or spread at a specific temperature.

Now entropy is commonly associated with a state of disorder, randomness, or uncertainty of the system and is widely used in various research fields for solving scientific tasks. Entropy formula, proposed by Shannon, is often used for solving multiple criteria optimization tasks to determine the objective weights of evaluation criteria.

Suppose that:

$$R=\left\{r_{1},{\;r}_{2},\ldots ,r_{n}\right\},$$

$$p_{j}=P\left(R=r_{j}\right),\;0<p_{j}<1\;,\;j=1,2,\ldots ,n,\overset{n}{\underset{j=1}{\sum }}p_{j}=1$$

is a source of information (discrete probability distribution) $\left\{\left(r_{1},p_{1}\right),\left(r_{2},p_{2}\right),\ldots ,\left(r_{n},p_{n}\right)\right\}.$ The classical measure of the entropy E(R), proposed by Shannon, is determined by the formula:

(3) $$E\left(R\right)=-\overset{n}{\underset{j=1}{\sum }}p_{j}\log p_{j}.$$

Here log is natural logarithm. The entropy E(R) acquires its maximum value E(R)=logn, when a source of information is distributed evenly: $p_{1}=p_{2}=\ldots =p_{n}=\frac{1}{n}.$ Note that the values of $r_{j},\;j=1,2,\ldots ,n\;$ are not required for calculation of entropy (3). Suppose that $R=\left\{r_{i},\;i=1,2,\ldots ,m\right\}$ is data array having n different values $r_{i}$. Let us count how many times each different value $r_{i}$ is repeated, and mark the corresponding frequencies $d_{i}:$ $\sum_{j=1}^{n}d_{i}=m,{\;p}_{i}=\frac{d_{i}}{m}.$ The data array R can then be treated as a particular source of information and its entropy calculated according to Formula (3).

When R is a result of certain measurements, the entropy calculated in this way may be sensitive to small changes in the data. For example, data array $R_{1}=\left\{0.24,\;0.25,\;0.29,\;0.31,\;0.32,\;0.85,\;0.86,\;0.87,\;0.88,\;0.89\right\}$ has all different $r_{i}$ values, therefore $d_{i}=1,{\;p}_{i}=\frac{1}{10},\;i=1,2,\ldots ,10$ and entropy acquires its maximum value $E\left(R_{1}\right)=\log10\approx 2.303$. If the measurement accuracy is ±0.5, we round the values and obtain a discrete probability distribution in Table 6:

Entropy in this case will be equal to

$$E\left(R_{1}^{*}\right)=-(0.2\;\log0.2+0.3\;\log0.3\;+0.3\;\log0.3\;+0.2\;\log0.2\;)\approx 1.336\;$$

and is significantly lower than $E\left(R_{1}\right)$: $\frac{E\left(R_{1}^{*}\right)}{E\left(R_{1}\right)}\approx 0.593.$

The idea of the WEBIRA method is to solve optimization problems minimizing disagreement between the weighted sums:

$$S_{X}^{\left(j\right)}=\overset{n_{x}}{\underset{i=1}{\sum }}w_{X_{i}}x_{i}^{\left(j\right)},{\;S}_{Y}^{\left(j\right)}=\overset{n_{y}}{\underset{i=1}{\sum }}w_{Y_{i}}y_{i}^{\left(j\right)}.$$

Here $x_{i}^{\left(j\right)}\left({\;y}_{i}^{\left(j\right)}\right)$ denote the normalized response of the alternative j to the criterion i. Weight coefficients $w_{X_{i}},{\;w}_{Y_{i}}$ must satisfy inequalities that correspond the identified priorities of indicators:

(4) $$w_{X_{1}}\geq w_{X_{2}}\geq \ldots \geq w_{X_{n_{x}}},{\;w}_{Y_{1}}\geq w_{Y_{2}}\geq \ldots \geq w_{Y_{n_{y}}\;.}$$

So, $X_{1}\;$ is the most important indicator, $X_{n_{x}}$ is the least important indicator in the criteria group X, accordingly $Y_{1}$ and $Y_{n_{y}}$ are the most and the least important indicators in the criteria group Y. The priority of indicators $x_{i},{\;y}_{i}$ may be determined by external requirements, such as expert estimates. The entropy method sets the importance of criteria $x_{i}\;$ and $y_{i}$ in calculating entropies of the sources:

$$X_{i}=\left\{x_{i}^{\left(1\right)},x_{i}^{\left(2\right)},\ldots ,x_{i}^{\left(m\right)}\right\},{\;Y}_{i}=\left\{y_{i}^{\left(1\right)},y_{i}^{\left(2\right)},\ldots ,y_{i}^{\left(m\right)}\right\}.$$

Priorities (4) are set in descending order of entropy values. When all the values of $r_{i}^{\left(j\right)}\;,\;j=1,2,\ldots ,m$ are different, the classical entropy (3) will acquire maximum value logm. In this case, several optimization tasks need to be addressed and this reduces the effectiveness of the WEBIRA method. This encouraged the authors of this article to modify the entropy calculation algorithm and introduce the notion of interval entropy. Interval $\left[a,b\right],\;a=\underset{j=1,2,\ldots ,m}{\min}r_{i}^{\left(j\right)},\;b=\underset{j=1,2,\ldots ,m}{\max}r_{i}^{\left(j\right)}$ is divided into m parts of equal length and frequencies $d_{i}$ calculated. $d_{i}\;$ means how many values $r_{j}$ fall into the corresponding interval. It is clear that:

$$\overset{m}{\underset{j=1}{\sum }}d_{i}=m\;.$$

Denote $p_{i}=\frac{d_{i}}{m}$ and calculate entropy by Formula (3). For example, in the case of data source $R_{1}$, we have a division interval length: $h=\frac{b-a}{m}=\frac{0.89-0.24}{10}=0.065,{\;a}_{0}=a=0.24,{\;a}_{i}=a_{i-1}+h,\;b\;=0.89.$ Intermediate calculations for $R_{1}\;$ are presented in Table 7.

Entropy value is

$$E\left(R_{1}\right)=-(0.3\;\log0.3+0.2\;\log0.2\;+0.5\;\log0.5\;)\approx 1.030.\;$$

Note that the entropy calculated in this way can change quite significantly with small changes in the data. This is the shortcoming of classical entropy. For example, the source $R_{2}=\left\{0.24,\;0.25,\;0.29,\;0.30,\;0.32,\;0.85,\;0.86,\;0.87,\;0.88,\;0.89\right\}$, which differs from $R_{1}$ by only one value at 0.30 instead of 0.31, and $p_{1}=0.4,{\;p}_{2}=0.1,p_{3}=0.5,$ so that

$$E\left(R_{2}\right)=-(0.4\;\log0.4+0.1\;\log0.1\;+0.5\;\log0.5\;)\approx 0.943.\;$$

In this paper, we define the interval entropy as a function of the interval number k:

(5) $$E_{k}\left(R\right)=-\frac{1}{\log k}\overset{k}{\underset{i=1}{\sum }}p_{i}\log p_{i},\;$$

where the values $p_{i}=\frac{d_{i}}{n}\;$ and frequencies $d_{i}$ $\left(\sum_{i=1}^{k}d_{i}=n\right)$ are defined as before. Note that the values $E_{k}\left(R\right)$ are normalized by the multiplier $\frac{1}{\log k}\;$ and do not depend on the logarithm base. Changing the parameter k of the function (5) allows efficient comparison of different information sources. Let us look at two more examples of information sources:

$$R_{3}=\left\{0.1,\;0.2,\;0.3,\;0.4,\;0.5,\;0.6,\;0.7,\;0.8,\;0.9,1.0\right\},\;R_{4}=\left\{0.1,\;0.16,\;0.34,\;0.35,\;0.57,\;0.58,\;0.72,\;0.73,\;0.95,1.0\right\}.$$

When k=2, we obtain probability distributions for $R_{3}$ and $R_{4}\;$ in Table 8:

$$E_{2}\left(R_{3}\right)=1.0,{\;E}_{2}\left(R_{4}\right)=-\frac{0.4\;\log0.4+0.6\;\log0.6}{\log2}\approx 0.971.$$

When k=5, in Table 9 we get the same frequencies and the same interval entropy values for data sources R_3_ and R_4_:

$$E_{5}\left(R_{3}\right)={\;E}_{5}\left(R_{4}\right)=1.0.$$

In the article below, we use the averages of the interval entropies (5) to compare the entropies of the sources $X_{i},Y_{i}$:

(6) $$E_{2,4,8,16}\left(R\right)=\frac{1}{4}\left(E_{2}\left(R\right)+E_{4}\left(R\right)+E_{8}\left(R\right)+E_{16}\left(R\right)\right),$$

(7) $$E_{2,4,7,14}\left(R\right)=\frac{1}{4}\left(E_{2}\left(R\right)+E_{4}\left(R\right)+E_{7}\left(R\right)+E_{14}\left(R\right)\right).$$

When the data is uniformly distributed in an interval, dividing it into 2,4,8, etc. parts, each part should have similar data numbers. Thus, the closer the uniform distribution is to the information source data, the higher is the average (6). Since the amount of data in sources $X_{i},Y_{i}\;$ is not large (m=28), it should be noted that in the case of the uniform distribution, the amount of data in the division intervals should be equal to the divisor of the number (m=28), i.e., 2,4,7,14. This is the reason to use both Formulas (6) and (7).

### 3.3. The Procedure of the WEBIRA Method for Calculating Criteria Weights

There is a wide variety of strategies to calculate criteria weights for solving multiple-criteria decision-making (MCDM) problems. In research [26], systematic review of the methods of determining criteria weights is presented. Two main directions of such strategies are subjective and objective methods of criteria weighting. Subjective methods are based on experts’ evaluations, while objective methods often use entropy. An integrated method is applied in [27] for this purpose.

In this Section, the weight-balancing method WEBIRA is used for calculation of indicator weights. Recall that nine indicators were divided into two groups X and Y. Indicators $C_{3},C_{4},{\;C}_{5},{\;C}_{6},\;$ describing the energy consumed and its structure, entered the first criteria group X; meanwhile, indicators $C_{1},{\;C}_{2},{\;C}_{7},C_{8},{\;C}_{9},$ describing the social–economic levels of countries and the situations in education, were assigned to the second group Y. The initial decision-making matrix is:

$$R=\left(X|Y\right),\;X={\left(x_{i}^{\left(j\right)}\right)}_{m\times n_{x}},\;Y={\left(y_{i}^{\left(j\right)}\right)}_{m\times n_{y}},{\;n}_{x}+n_{y}=n.$$

Here, m=28 is the number of alternatives (EU countries); $n_{x}=4$ and $n_{y}=5$ are the numbers of indicators in the first and second groups correspondingly. We determined weighted sums for each alternative separately for X and Y group indicators:

$$S_{X}^{\left(j\right)}=\overset{n_{x}\;}{\underset{i=1}{\sum }}w_{x_{i}}x_{i}^{\left(j\right)},{\;S}_{Y}^{\left(j\right)}=\overset{n_{y}}{\underset{i=1}{\sum }}w_{y_{i}}y_{i}^{\left(j\right)},\;j=1,2,\ldots ,m,$$

and for arbitrary weight vectors:

$$W_{X}=\left(w_{x1},w_{x2},\ldots ,w_{{\mathrm{xn}}_{x}}\right),{\;W}_{Y}=\left(w_{y1},w_{y2},\ldots ,w_{{\mathrm{yn}}_{y}}\right),$$

satisfying the conditions:

(8) $$1\geq w_{x1}\geq w_{x2}\geq \ldots \geq w_{{\mathrm{xn}}_{x}}\geq 0,\;\;1\geq w_{y1}\geq w_{y2}\geq \ldots \geq w_{{\mathrm{yn}}_{y}}\geq 0$$

and:

(9) $$\overset{n_{x}\;}{\underset{i=1}{\sum }}w_{x_{i}}=\overset{n_{y}\;}{\underset{i=1}{\sum }}w_{y_{i}}=\;1.$$

Weight prioritization in Formula (8) must match the prioritization of indicators, established according to decreasing interval entropy values. For example, in 2017 we have the unique indicator’s prioritization (15); consequently, prioritization of weights may be as follows:

(10) $$1\geq w_{x_{4}}\geq w_{x_{3}}\geq w_{x_{5}}\geq w_{x_{6}}\geq 0,\;\;1\geq w_{y_{8}}\geq w_{y_{9}}\geq w_{y_{7}}\geq w_{y_{2}}\geq w_{y_{1}}\geq 0.$$

We denoted $R_{X}^{j}$ $(R_{Y}^{j}),\;j=1,2,\ldots ,m$ ranks of alternatives assigned by decreasing weighted sum $S_{X}^{\left(j\right)}\;\left(S_{Y}^{\left(j\right)}\right)\;$ values. Our purpose was to calculate weights $W_{X}$ and $W_{Y,}$ which minimize disagreements between two EU countries’ prioritizations: only by X and by Y indicators. In the papers [28,29,30,31] the weights satisfying conditions (8)–(9) were calculated by solving the optimization problem:

(11) $$S\left(W_{X},W_{Y}\right)=\underset{W_{X},W_{Y}}{\min}\overset{m}{\underset{j=1}{\sum }}{\left(S_{X}^{\left(j\right)}-S_{Y}^{\left(j\right)}\right)}^{2}.$$

Unfortunately, the goal function (11) is not acceptable for our data due to insufficient sensitivity. In this article, the two-component goal function is applied: sum of the differences of weighted sums’ absolute values, and sum of the differences of ranks, calculated separately in each group of indicators.

(12) $$F\left(W_{X},W_{Y}\right)=\underset{W_{X},W_{Y}}{\min}(\;\frac{1}{m}\;\overset{m}{\underset{j=1}{\sum }}|S_{X}^{\left(j\right)}-S_{Y}^{\left(j\right)}|+\overset{m}{\underset{j=1}{\sum }}|R_{X}^{\left(j\right)}-R_{Y}^{\left(j\right)}|).$$

### 3.4. Algorithm for Criteria Weight Calculation

In this research, the following algorithm is implemented to calculate criteria weights:

(i) Algorithm parameters are determined:
  $$10^{-3}<\varepsilon <10^{-1},10^{2}<\max_{\mathrm{iter}}<10^{6}.$$
(ii) The initial vector of weights $w^{0}$ satisfying conditions (8) is chosen:
  $$w^{0}=(w_{x_{\left(1\right)}}^{0},w_{x_{\left(2\right)}}^{0},\ldots ,w_{x_{\left(n_{x}\right)}}^{0};{\;w}_{y_{\left(1\right)}}^{0},w_{y_{\left(2\right)}}^{0},\ldots ,w_{y_{\left(n_{y}\right)}}^{0}).$$
(iii) Randomly selected vector of direction:
  $$\Delta w=\left({\Delta w}_{x_{\left(1\right)}},{\Delta w}_{x_{\left(2\right)}},\ldots ,{\Delta w}_{x_{\left(n_{x}\right)}};{\;\Delta w}_{y_{\left(1\right)}},{\Delta w}_{y_{\left(2\right)}},\ldots ,{\Delta w}_{y_{\left(n_{y}\right)}}\right).$$
(iv) Vector $w^{1}=w^{0}+\varepsilon \Delta w$ is calculated. If it does not satisfy conditions (8), the following corrections must be done:
  (a) If $w_{x,y_{\left(i\right)}}^{1}<0,$ change $w_{x,y_{\left(i\right)}}^{1}=0$;
  (b) $w_{x,y_{\left(i\right)}}^{1}<w_{x,y_{\left(i+1\right)}}^{1}$, change $w_{x,y_{\left(i\right)}}^{1}=w_{x,y_{\left(i+1\right)}}^{1}$;
  (c) If $s=\sum_{i=1}^{n_{x},n_{y}}w_{x,y_{\left(i\right)}}^{1}\neq 1$, change $w_{x,y_{\left(i\right)}}^{1}=\frac{w_{x,y_{\left(i\right)}}^{1}}{s}$.
(v) The number of iterations iter is calculated.
(vi) If $\mathrm{iter}>\max_{\mathrm{iter}}$, then the algorithm is finishing calculations.
(vii) If F(w^1^) > F(w^0^) then go to point (iii) of the algorithm, or else assign w^0^ = w^1^ and go to point (iii) of the algorithm.

## 4. Results

### 4.1. Prioritization of Europe-28 Headline Indicators

Classical entropy values E(R) of indicators $C_{1}-C_{9}$, and interval entropies $E_{2},{\;E}_{4},{\;E}_{7},{\;E}_{8},{\;E}_{14},{\;E}_{16},$ as well as their average values $E_{2,4,7,14},{\;E}_{2,4,8,16}$ were calculated using normalized data. The results of calculations for entropies of Europe 2020 headline indicators in 2016 are given in Table 10.

Note that prioritization is calculated separately for the two groups of indicators, X and Y. For 2016 data we obtained two prioritizations. The first one is according to decreasing entropy $E_{2,4,8,16}\;$ values:

(13) $$X:{\;C}_{4}≻C_{3}≻C_{5}≻C_{6},\;Y:{\;C}_{8}≻C_{2}≻C_{1}≻C_{9}≻C_{7}.$$

Second prioritization has been set according to decreasing entropy $E_{2,4,7,14}\;$ values:

(14) $$X:{\;C}_{4}≻C_{3}≻C_{5}≻C_{6},\;Y:{\;C}_{2}≻C_{8}≻C_{9}≻C_{1}≻C_{7}.$$

By analogously calculating entropies for 2017 and 2018 data, we obtained the results written in Table 11 and Table 12.

Data in Table 11 for 2017 leads in the unique indicator’s prioritization:

(15) $$X:{\;C}_{4}≻C_{3}≻C_{5}≻C_{6},\;Y:{\;C}_{8}≻C_{9}≻C_{7}≻C_{2}≻C_{1}.$$

Entropies $E_{2,4,8,16}$ and $E_{2,4,7,14}$ in Table 12 give two different prioritizations of indicators for 2018, accordingly:

(16) $$X:{\;C}_{3}≻C_{4}≻C_{5}≻C_{6},\;Y:{\;C}_{9}≻C_{2}≻C_{7}≻C_{8}≻C_{1},$$

(17) $$X:{\;C}_{3}≻C_{4}≻C_{5}≻C_{6},\;Y:{\;C}_{9}≻C_{8}≻C_{7}≻C_{2}≻C_{1}.$$

Note, that in the first group of indicators X, priorities change minimally in subsequent years—with only $C_{3}$ and $C_{4}\;$ changing places. So, the main important indicators in group X are $C_{4}$ (share of renewable energy in gross final energy consumption) in 2016–2017 and $C_{3}$ (greenhouse gas emission) in 2018. In the second group Y the main important are $C_{8}$ (tertiary education attainment) in 2016–2017 and $C_{9}$ (people at risk of poverty and social exclusion). The main important indicators identify areas with the greatest inequalities between countries.

### 4.2. Results of Criteria Weight Calculation

The algorithm described in Section 3.4 was applied to the initial decision-making matrices data for 2016–2018 submitted in Table 2, Table 3 and Table 4. Values of indicators were preliminarily normalized by the Formulas (1) and (2). For 2016 data we have two prioritizations. Accordingly, two weight vectors were obtained. The first one matched criteria prioritization (13) according to decreasing entropy $E_{2,4,8,16}\;$ values:

$$X:{\;w}_{x_{4}}\geq w_{x_{3}}\geq w_{x_{5}}\geq w_{x_{6}},\;Y:w_{y_{8}}\geq w_{y_{2}}\geq w_{y_{1}}\geq w_{y_{9}}\geq w_{y_{7}}.\;$$

Weight values and the goal function $F\left(W_{X},W_{Y}\right)\;$ value were obtained as follows:

$$w_{x_{4}}=0.8637,{\;w}_{x_{3}}=0.1264,{\;w}_{x_{5}}=0.0099,w_{x_{6}}=0;\;w_{y_{8}}=0.4286,{\;w}_{y_{2}}=0.2009,{\;w}_{y_{1}}=0.2009,w_{y_{9}}=0.0848,w_{y_{7}}=0.0848;\;F\left(W_{X}^{0},W_{Y}^{0}\right)=224.076686.$$

The second solution of the optimization problem (12) corresponds with criteria prioritization (14) according to decreasing entropy $E_{2,4,7,14}\;$ values:

$$X:{\;w}_{x_{4}}\geq w_{x_{3}}\geq w_{x_{5}}\geq w_{x_{6}},\;Y:w_{y_{2}}\geq w_{y_{8}}\geq w_{y_{9}}\geq w_{y_{1}}\geq w_{y_{7}}.\;$$

Weight values and the goal function value are:

$$w_{x_{4}}=0.8173,{\;w}_{x_{3}}=0.1816,{\;w}_{x_{5}}=0.0011,w_{x_{6}}=0;\;w_{y_{2}}=0.9290,{\;w}_{y_{8}}=0.0182,{\;w}_{y_{9}}=0.0182,w_{y_{1}}=0.0173,w_{y_{7}}=0.0173;\;F\left(W_{X}^{0},W_{Y}^{0}\right)=204.109514.$$

The goal function value is lower for the second solution, so we choose it as an optimal solution for 2016.

Criteria weight prioritization for 2017 is unique (see (15)):

$$X:{\;w}_{x_{4}}\geq w_{x_{3}}\geq w_{x_{5}}\geq w_{x_{6}},\;Y:w_{y_{8}}\geq w_{y_{9}}\geq w_{y_{7}}\geq w_{y_{2}}\geq w_{y_{1}}.\;$$

Weight values and the goal function value are:

$$w_{x_{4}}=0.6719,{\;w}_{x_{3}}=0.3281,{\;w}_{x_{5}}=0,w_{x_{6}}=0;\;w_{y_{8}}=0.4019,{\;w}_{y_{9}}=0.1572,{\;w}_{y_{7}}=0.1572,w_{y_{2}}=0.1419,w_{y_{1}}=0.1419;\;F\left(W_{X}^{0},W_{Y}^{0}\right)=230.062190.$$

There are also two criteria prioritizations for 2018. The weights matching the first prioritization (16) are:

$$X:{\;w}_{x_{3}}\geq w_{x_{4}}\geq w_{x_{5}}\geq w_{x_{6}},\;Y:w_{y_{9}}\geq w_{y_{2}}\geq w_{y_{7}}\geq w_{y_{8}}\geq w_{y_{1}},$$

$$w_{x_{3}}=0.5745,{\;w}_{x_{4}}=0.4135,{\;w}_{x_{5}}=0.0099,w_{x_{6}}=0.0021;\;w_{y_{9}}=0.3482,{\;w}_{y_{2}}=0.3086,{\;w}_{y_{7}}=0.2385,w_{y_{8}}=0.1042,w_{y_{1}}=0.0005;\;F\left(W_{X}^{0},W_{Y}^{0}\right)=220.053163$$

and the second prioritization (17) are:

$$X:{\;w}_{x_{3}}\geq w_{x_{4}}\geq w_{x_{5}}\geq w_{x_{6}},\;Y:w_{y_{9}}\geq w_{y_{8}}\geq w_{y_{7}}\geq w_{y_{2}}\geq w_{y_{1}},\;$$

$$w_{x_{3}}=0.5759,{\;w}_{x_{4}}=0.4240,{\;w}_{x_{5}}=0.0001,w_{x_{6}}=0;\;w_{y_{9}}=0.3384,{\;w}_{y_{8}}=0.2206,{\;w}_{y_{7}}=0.2205,w_{y_{2}}=0.2205,w_{y_{1}}=0;\;F\left(W_{X}^{0},W_{Y}^{0}\right)=228.053106.$$

Goal function value is lower for the first solution, so we choose it as an optimal solution for 2018. Table 13 summarizes information about indicator weights that minimize the value of goal function (12).

In 2016–2017, the most informative X group indicator is C_4_ (share of renewable energy in gross final energy consumption), then goes C_3_ (greenhouse gas emission). In 2018, C_3_ and C_4_ swap places, C_3_ becomes most informative. The least informative X group indicators in 2016–2018 are C_5_, C_6_ (primary energy consumption, final energy consumption).

Y group indicator C_2_ (gross domestic expenditure on research and development) has the highest priority in 2016, C_8_ (tertiary education attainment) in 2017, and C_9_ (people at risk of poverty and social exclusion) in 2018. In most cases, the least important indicators are C_1_ (employment rate) and C_7_ (early leavers from education and training).

Note that the distribution of weights is more even in 2017–2018 compared to 2016 when the most informative indicators have extremely high values.

### 4.3. Ranking the Alternatives

The alternatives ranking procedure was carried out taking into account both ranks assigned to the alternatives in separate groups of indicators X and Y. The α-cuts procedure is elaborated for this purpose.

Suppose that initial decision-making matrix is a concatenation of two matrices X and Y:

$$R=\left(X|Y\right),\;X={\left(x_{i}^{\left(j\right)}\right)}_{m\times n_{x}},\;Y={\left(y_{i}^{\left(j\right)}\right)}_{m\times n_{y}},{\;n}_{x}+n_{y}=n.$$

Here, m = 28 is the number of alternatives (EU countries); n_x_ and n_y_ are numbers of indicators in the first and second indicator groups. Determine weighted sums

$$S_{X}^{\left(j\right)}\left(W_{X}^{0}\right)=\overset{n_{x}\;}{\underset{i=1}{\sum }}w_{x_{i}}^{0}x_{i}^{\left(j\right)},{\;S}_{Y}^{\left(j\right)}\left(W_{Y}^{0}\right)=\overset{n_{y}}{\underset{i=1}{\sum }}w_{y_{i}}^{0}y_{i}^{\left(j\right)},\;j=1,2,\ldots ,m,\;$$

where $W_{X}^{0}=\left(w_{x_{1}}^{0},\;\ldots ,w_{x_{n_{x}}}^{0}\right)$ and $W_{Y}^{0}=\left(w_{y_{1}}^{0},\;\ldots ,w_{x_{n_{y}}}^{0}\right)$ are solutions of optimization problem (12) satisfying the conditions (8–9). Let α be a positive number and 0<α<1. Denote $A_{\alpha }$–the set of alternatives $j^{\left(1\right)},{\;j}^{\left(2\right)},\ldots ,j^{\left(k_{\alpha }\right)}$ for which two inequalities are fulfilled:

(18) $$S_{X}^{\left(j\right)}\left(W_{X}^{0}\right)\geq \alpha ,{\;S}_{Y}^{\left(j\right)}\left(W_{Y}^{0}\right)\geq \alpha ,\;j\in A_{\alpha }.$$

We call $A_{\alpha }\;$ the α-cut of the set of alternatives  {1,2,…,m}. The α-cut accumulates the best alternatives, taking into account both groups of indicators. It is clear that $A_{1}$ is an empty set. By lowering the threshold α, the number of alternatives that satisfy inequalities (18) increases. Consequently, the number of alternatives in A_α_ increases. Finally, α-cut A_0_ contains all m alternatives. Let the initial value of α be equal to 1. By gradually reducing the value of α we will obtain α-cuts containing respectively 1,2,…,m alternatives and assign them corresponding ranks. In other words, ranks are assigned to alternatives according to decreasing values of:

$$\min\left(S_{X}^{\left(j\right)}\left(W_{X}^{0}\right),{\;S}_{Y}^{\left(j\right)}\left(W_{Y}^{0}\right)\right)$$

The results of the α-cut procedure—ranks of EU-28 countries according to interrelation between indicators of X and Y groups in 2016–2018—are given in Table 14, Table 15 and Table 16. Respective weights $(W_{X}^{0}$,${\;W}_{Y}^{0})\;$ solutions of the optimization problem (12) satisfying conditions (8–9) are presented in Table 13. In the third column of Table 14, Table 15 and Table 16, there are countries that enter the α-cut at the respective α level. In 2016 (see Table 14), the first country entering α-cut $A_{0.3616}$ is Sweden. Inequalities (18) are satisfied for α = 0.3616. Rank 1 is assigned to this European country. The second country is Austria, having rank 2; this country together with Sweden enters $A_{0.3477}$. Continuing this process in the last step, we include Romania in α-cut $A_{0.0771}$ and assign rank 28. This α-cut involves all 28 European countries.

### 4.4. Countries Clustering Solution

**Figure 2:**
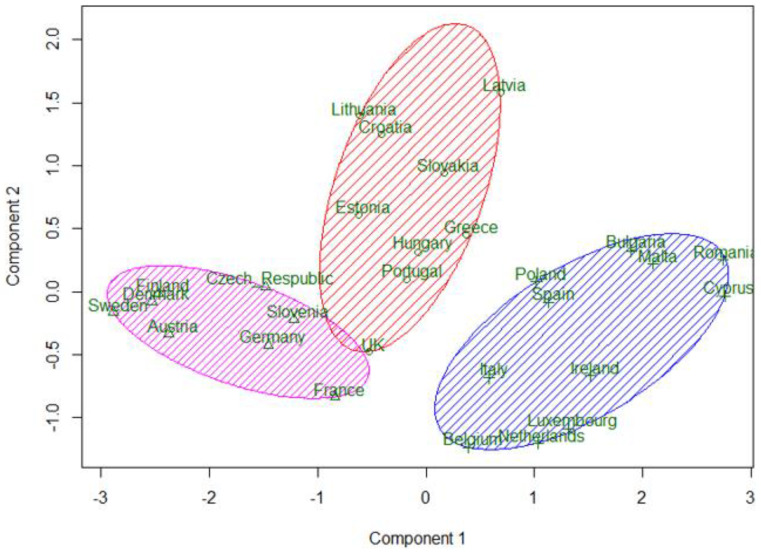
k-means three-clusters solution obtained by the weight-balancing indicator ranks accordance (WEBIRA) method from 2016–2018 European countries’ ranking.

**Figure 3:**
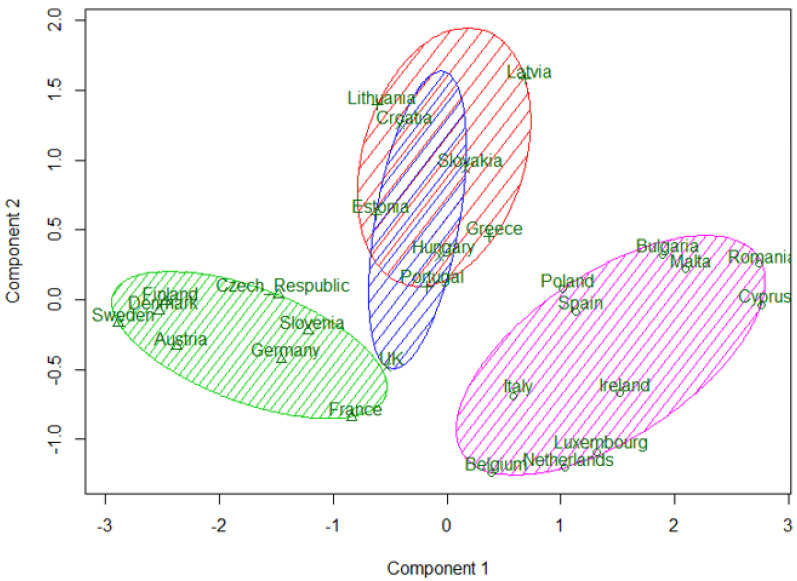
k-means four-clusters solution obtained by the WEBIRA method from 2016–2018 European countries’ ranking.

A k-means clustering procedure was applied to the EU-28 countries. Calculations were performed with the help of software package R. In the first experiment, the clustering procedure was applied to nine variables—headline indicators. The obtained result distinguished two clusters of strong and weak countries, and a third cluster was formed from only three countries—Luxembourg, Netherlands, and Belgium. Being economically strong, these countries have not shown a major breakthrough in implementing the Europe 2020 strategy. However, the aim of this study was to create three clusters based on the WEBIRA method of ranking results of European countries and to compare these clusters with the grouping of countries into three groups formed in the study by Fedajev et al. [4]. Therefore, in the second experiment, Member States were clustered by three variables—the WEBIRA ranking results obtained for 2016–2018 presented in the last columns of Table 14, Table 15 and Table 16. This clustering solution is compared with assigning countries to three groups in [4]. In Fedajev et al. [4], with final ranking implemented by the MULTIMOORA method, countries were assigned to the core, semi-periphery, and periphery groups, which are analogues of Clusters 1, 2, and 3. Both clustering results presented in comparison Table 17. In general, countries’ categorization results in [4] coincide with three clusters of k-means clustering (see Table 17). However, there were discrepancies that are marked with a gray background. In current research, Croatia and Lithuania belong to the middle group (Cluster 2), while in [4], these countries are categorized to the core group (Cluster 1). Poland and Ireland belong to the weakest group (Cluster 3) according to our classification and to the semi-periphery group (Cluster 2) by [4]. By summarizing the ranking results in 2016–2018, Germany is in Cluster 1 (the most advanced group) and in Cluster 2 according to [4]. The three-clusters solution obtained by the WEBIRA method is depicted in Figure 2, and the four-clusters solution—in Figure 3. Here we can see three and four clusters against the first two principal components. The first principal component separates very well all three clusters in Figure 2; meanwhile, the second principal component separates well only Cluster 2 from other clusters, but cannot separate Clusters 1 and 3. The four-clusters solution leaves the same clusters of the most advanced and weakest countries and divides the middle cluster into two parts. One part includes the Baltic countries—Estonia, Latvia, Lithuania—and two south European countries—Greece and Portugal (Cluster 4). The second part includes Croatia, Slovakia, Hungary, and the United Kingdom (Cluster 2). Thus, the four-clusters solution confirms the stability of clusters of the strongest and weakest countries.

It is worth mentioning that the first two principal components separate the initial variables very well and accumulate 95.4% of initial data variability. Principal components are linear combinations of the initial variables. The following expressions of the main components were obtained after non-orthogonal oblimin rotation:

$$F_{1}=-0.035R_{2016}+0.589R_{2017}+0.473R_{2018};$$

$$F_{2}=0.973R_{2016}-0.136R_{2017}+0.145R_{2018}.$$

**Figure 4:**
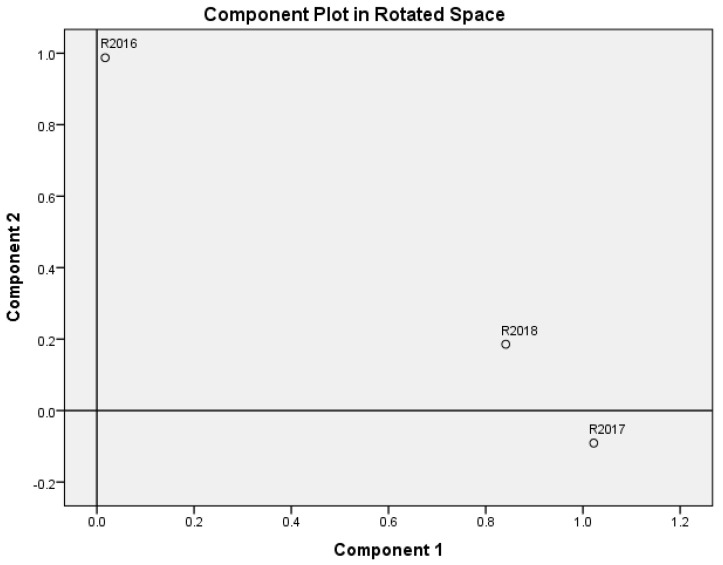
Variables $R_{2016},R_{2017},{\;R}_{2018}$ in rotated space of principal components $F_{1}$ and $F_{2}$.

Here $F_{1}$ and $F_{2}$ are first two principal components, $R_{2016}-R_{2018}$ are WEBIRA rankings of EU-28 countries. So, variables $R_{2017}$ and $R_{2018}$ have the highest weights in $F_{1}$; meanwhile, $F_{2}$ consists mainly of variable $R_{2016}.$ In Figure 4, variables $R_{2016}-R_{2018}$ are represented in rotated component space. The figure confirms that the first principal component is related to $R_{2017}$ and $R_{2018}$, and the second component to the variable $R_{2018}$.

## 5. Discussion

The set of headline indicators that are the main criteria for evaluating countries was subjectively divided into two logically explained groups. However, in some cases it could be possible to apply objective methods, such as the principal components method, for this purpose. On the other hand, this method is not universal, as it is known that it is not suitable for all data sets. The principal components method is not applicable if correlations between variables are not high enough. In such cases we could not be able to unambiguously assign variables to factors. The same problem has occurred with the Europe 2020 strategy headline indicators for 2016–2018. Attempts to distinguish principal components using headline indicators were unsuccessful.

In this paper, interval entropy has been applied to prioritize indicators within groups. Classical entropy has an undesirable property—after making small changes to the original data, the value of entropy changes drastically. It was shown that interval entropy, unlike classical entropy, responds adequately to small changes in baseline data. This allows the indicators to be prioritized more correctly.

The WEBIRA method was chosen to calculate the weights of the indicators. The WEBIRA method has an advantage in its weight-balancing procedure, that assigns weights to indicators by solving the optimization problem—the difference between ranking results according to both groups of indicators have to be minimized. Consequently, calculated weights minimize disagreement between two EU countries’ prioritizations–only by X and only by Y indicators. There is a wide variety of MCDM methods that can be used to calculate criteria weights. This step is very important in the whole analysis, since the ranking of countries depends crucially on how the weights are assigned.

Traditionally, MCDM methods construct a goal function, which is used to rank countries. In this research, the α-cuts procedure was used for ranking European countries by interdependence between two groups of indicators. This choice, in our opinion, is logical because it takes into account the rankings of countries according to separate groups of indicators, and aggregates and generalizes these results.

Finally, the k-means clustering procedure, based on 2016–2018 ranking results, made it possible to identify groups of countries that had achieved different results in implementing the Europe 2020 strategy. It should be noted that clustering by using the initial variables did not yield a satisfactory result. Despite the choice of a three-cluster solution, almost all countries were classified into two clusters, with only Luxembourg, the Netherlands, and Belgium in the third.

The proposed methodology could be applied to many tasks that require optimization problems according to a heterogeneous set of conflicting criteria. Interval entropy allows more precise ranking of evaluation criteria according to their importance. The α-cuts ranking technology evaluates all alternatives according to the interdependence of several groups of criteria.

## 6. Conclusions and Future Research

In our study, we raised the objective of ranking European Union member states in the implementation of the Europe 2020 strategy on the basis of the results achieved in 2016–2018 and propose a clustering solution of the countries based on 2016–2018 rankings.

Table 14, Table 15 and Table 16 show the final ranking of European countries according to the interrelation between consumed energy structure and its quantity (X group of indicators) and social–economic level of countries and situation in education (Y group of indicators). The rating leaders, Sweden, Austria, Denmark, and Finland, don’t change during the three years 2016–2018. It is obvious that these European countries are distinguished by their responsible approach to the sustainable use of natural resources; these countries are also more successful in solving education system problems and challengers; and their economies are strong. The outsiders of this rating are Cyprus, Romania, Malta, and Bulgaria. In 2016–2018 they were not as successful as other countries in meeting national targets.

Some countries have made significant progress over a three-year period. For example, Croatia’s rank changed from 22nd in 2016, to 10th in 2017, and to 7th in 2018. The Czech Republic’s rank rose from 9th in 2016–2017, to 5th in 2018; Lithuania—from 21st place in 2016 to 5th in 2017, and 10th in 2018. Great progress has been demonstrated by Slovakia: 2016—23rd, 2017—16th, and 2018—8th. But not all countries were such successful. The situation got worse over a three-year period for Ireland, Spain, Italy, and Cyprus. Two countries—new Members of the European Union, Slovenia and the Czech Republic, took their places between leaders in the Europe 2020 strategy implementation. However, Slovenia is lagging on greenhouse gas emission, the Czech Republic on share of renewable energy in gross final energy consumption, and both countries stand behind on gross domestic expenditure on research and development. Considering that there is very little time left until the end of the strategy implementation period, it is doubtful that this indicator can reach the target value of three percent in these countries.

k-means clustering results were compared to formal classification of EU-28 members into three groups: core, semi-periphery, and periphery obtained in [4] (investigation is based on the results in 2016). In general, the classification results are very similar, but there are also discrepancies shown in Table 17.

Looking at the perspective of future research, the authors believe that it would be appropriate to form three groups of logically related indicators instead of two groups: indicators that describe the amount of energy consumed and its structure as well as the exhaust gas emissions; social–economic indicators; and indicators describing the situations in education. It would be interesting to compare the ranking results of European countries for the cases of two and three groups of indicators.

Another possible direction for further research could be the analysis of panel data, when the raw data is treated both as a data frame and as time series data. In this way, it would become possible to model available data sequences and make predictions.

Numerical analysis of three entropy-based methods in [31] revealed that WEBIRA demonstrates higher efficiency comparing to other entropy-based methods. However, we did not perform similar comparative analysis on the WEBIRA and MULTIMOORA methods. Such a study could be carried out in the future work.

In our future research, we are going forward to look for important emerging application areas of our methodology. MCDM methods have a very wide range of applications—in economics, technology, construction, logistics, energy, the supplier/provider/material selection, etc. We plan to adapt our methodology to address public health issues, such as comparing the effectiveness of vaccines, drugs and treatment methodologies.

## Figures and Tables

**Table 1:** EU-28 countries Europe 2020 headline indicator values in 2016–2018 and their targets.

	Indicator	2016	2017	2018	Target
C_1_	Employment Rate, age group 20–64 (%)	71.10	72.20	73.20	75
C_2_	Gross Domestic Expenditure on Research and Development (% of GDP)	2.04	2.08	2.11	3
C_3_	Greenhouse Gas Emission, Base Year 1990, Index (1990 = 100)	77.92	78.38	76.76	80
C_4_	Share of Renewable Energy in Gross Final Energy Consumption (%)	16.995	17.474	17.98	20
C_5_	Primary Energy Consumption (million tonnes of oil equivalent (TOE) )	1544.93	1562.40	1551.92	1483
C_6_	Final Energy Consumption (million TOE)	1110.02	1122.93	1124.14	1086.00
C_7_	Early Leavers From Education and Training (% of the Population aged 18–24)	10.7	10.5	10.5	10
C_8_	Tertiary Education Attainment, Age Group 30–34 (%)	39.2	39.9	40.7	40
C_9_	People at Risk of Poverty and Social Exclusion (Million People)	118.06	112.93	109.87	96.2

Source: Eurostat (Europe 2020 headline indicators [21]).

**Table 2:** EU-28 countries’ Europe 2020 headline indicator values in 2016.

	C_1_	C_2_	C_3_	C_4_	C_5_	C_6_	C_7_	C_8_	C_9_
Belgium	67.70	2.52	81.96	8.71	4.35	3.22	8.80	45.60	20.90
Bulgaria	67.70	0.77	58.52	18.76	2.47	1.35	13.80	33.80	40.40
Czech Republic	76.70	1.68	66.06	14.93	3.79	2.35	6.60	32.80	13.30
Denmark	76.00	3.09	73.74	31.84	3.08	2.56	7.50	46.50	16.80
Germany	78.60	2.94	74.17	14.89	3.62	2.64	10.30	33.20	19.70
Estonia	76.60	1.25	48.98	28.68	4.48	2.16	10.90	45.40	24.40
Ireland	71.40	1.17	113.34	9.26	3.09	2.45	6.00	54.60	24.40
Greece	56.20	0.99	89.74	15.39	2.12	1.55	6.20	42.70	35.60
Spain	63.90	1.19	116.51	17.43	2.57	1.77	19.00	40.10	27.90
France	70.00	2.22	85.44	15.68	3.60	2.24	8.80	43.70	18.20
Croatia	61.40	0.86	76.15	28.27	1.92	1.58	2.80	29.30	27.90
Italy	61.60	1.37	85.80	17.42	2.44	1.91	13.80	26.20	30.00
Cyprus	68.70	0.52	151.00	9.86	2.86	2.09	7.60	53.40	27.70
Latvia	73.20	0.44	43.55	37.14	2.18	1.94	10.00	42.80	28.50
Lithuania	75.20	0.84	42.77	25.61	2.09	1.77	4.80	58.70	30.10
Luxembourg	70.70	1.30	87.98	5.44	7.20	7.01	5.50	54.60	19.80
Hungary	71.50	1.19	65.48	14.32	2.41	1.81	12.40	33.00	26.30
Malta	71.10	0.57	83.84	6.21	1.58	1.29	19.20	32.00	20.30
Netherlands	77.10	2.00	91.57	5.83	3.81	2.93	8.00	45.70	16.70
Austria	74.80	3.12	103.05	33.37	3.67	3.23	6.90	40.10	18.00
Poland	69.30	0.96	84.56	11.27	2.50	1.75	5.20	44.60	21.90
Portugal	70.60	1.28	115.32	30.87	2.10	1.57	14.00	34.60	25.10
Romania	66.30	0.48	46.29	25.03	1.55	1.13	18.50	25.60	38.80
Slovenia	70.10	2.01	94.69	21.29	3.17	2.36	4.90	44.20	18.40
Slovakia	69.80	0.79	57.72	12.03	2.83	1.92	7.40	31.50	18.10
Finland	73.40	2.72	83.16	39.01	5.91	4.59	7.90	46.10	16.60
Sweden	81.20	3.25	76.99	53.37	4.61	3.25	7.40	51.00	18.30
United Kingdom	77.50	1.66	63.76	8.98	2.74	2.05	11.20	48.10	22.20

Source: Eurostat (Europe 2020 headline indicators [21]).

**Table 3:** EU-28 countries’ Europe 2020 headline indicator values in 2017.

	C_1_	C_2_	C_3_	C_4_	C_5_	C_6_	C_7_	C_8_	C_9_
Belgium	68.50	2.66	82.14	9.06	4.32	3.18	8.90	45.90	20.60
Bulgaria	71.30	0.74	60.88	18.70	2.58	1.39	12.70	32.80	38.90
Czech Republic	78.50	1.79	65.56	14.80	3.81	2.41	6.70	34.20	12.20
Denmark	76.60	3.05	70.67	34.72	3.11	2.58	8.80	48.20	17.20
Germany	79.20	3.07	73.22	15.47	3.61	2.65	10.10	34.00	19.00
Estonia	78.70	1.28	52.26	29.13	4.29	2.18	10.80	48.40	23.40
Ireland	73.00	1.24	113.29	10.59	3.01	2.45	5.00	54.50	22.70
Greece	57.80	1.13	93.62	16.95	2.15	1.56	6.00	43.70	34.80
Spain	65.50	1.21	121.49	17.56	2.70	1.82	18.30	41.20	26.60
France	70.60	2.21	86.35	16.01	3.58	2.22	8.80	44.40	17.00
Croatia	63.60	0.86	78.71	27.28	2.01	1.67	3.10	28.70	26.40
Italy	62.30	1.37	85.05	18.27	2.46	1.90	14.00	26.90	28.90
Cyprus	70.80	0.55	155.75	10.49	2.96	2.19	8.50	55.90	25.20
Latvia	74.80	0.51	43.94	39.02	2.29	2.06	8.60	43.80	28.20
Lithuania	76.00	0.90	43.24	26.04	2.16	1.88	5.40	58.00	29.60
Luxembourg	71.50	1.27	90.88	6.29	7.26	7.08	7.30	52.70	21.50
Hungary	73.30	1.33	68.25	13.52	2.50	1.89	12.50	32.10	25.60
Malta	73.00	0.58	93.45	7.27	1.76	1.35	17.70	33.50	19.30
Netherlands	78.00	1.98	90.78	6.46	3.81	2.94	7.10	47.90	17.00
Austria	75.40	3.05	106.17	33.14	3.74	3.26	7.40	40.80	18.10
Poland	70.90	1.03	87.70	10.96	2.61	1.87	5.00	45.70	19.50
Portugal	73.40	1.32	123.78	30.61	2.21	1.61	12.60	33.50	23.30
Romania	68.80	0.50	47.39	24.45	1.65	1.18	18.10	26.30	35.70
Slovenia	73.40	1.87	93.47	21.06	3.26	2.40	4.30	46.40	17.10
Slovakia	71.10	0.89	59.31	11.47	2.97	2.05	9.30	34.30	16.30
Finland	74.20	2.73	79.59	40.92	5.83	4.59	8.20	44.60	15.70
Sweden	81.80	3.37	76.52	54.20	4.65	3.23	7.70	51.30	17.70
United Kingdom	78.20	1.68	62.70	9.73	2.69	2.03	10.60	48.20	22.00

Source: Eurostat (Europe 2020 headline indicators [21]).

**Table 4:** EU-28 countries’ Europe 2020 headline indicator values in 2018.

	C_1_	C_2_	C_3_	C_4_	C_5_	C_6_	C_7_	C_8_	C_9_
Belgium	69.70	2.76	82.67	9.42	4.11	3.19	8.60	47.60	20.00
Bulgaria	72.40	0.76	57.16	20.53	2.60	1.41	12.70	33.70	32.80
Czech Republic	79.90	1.93	64.82	15.15	3.81	2.39	6.20	33.70	12.20
Denmark	77.50	3.03	70.69	35.71	3.11	2.59	10.40	48.40	17.00
Germany	79.90	3.13	70.44	16.48	3.52	2.60	10.30	34.90	18.70
Estonia	79.50	1.40	49.98	30.00	4.68	2.24	11.30	47.20	24.40
Ireland	74.10	1.15	113.60	11.06	3.01	2.54	5.00	56.30	21.10
Greece	59.50	1.18	90.84	18.00	2.09	1.48	4.70	44.30	31.80
Spain	67.00	1.24	119.74	17.45	2.67	1.86	17.90	42.40	26.10
France	71.30	2.20	83.10	16.59	3.57	2.19	8.70	46.20	17.40
Croatia	65.20	0.97	75.23	28.02	1.99	1.67	3.30	34.10	24.80
Italy	63.00	1.39	84.41	17.78	2.43	1.93	14.50	27.80	27.30
Cyprus	73.90	0.55	153.81	13.88	2.95	2.15	7.80	57.10	23.90
Latvia	76.80	0.64	45.95	40.29	2.42	2.16	8.30	42.70	28.40
Lithuania	77.80	0.94	42.64	24.45	2.25	1.98	4.60	57.60	28.30
Luxembourg	72.10	1.21	94.16	9.06	7.41	7.23	6.30	56.20	21.90
Hungary	74.40	1.53	67.82	12.49	2.50	1.90	12.50	33.70	19.60
Malta	75.50	0.57	96.14	7.98	1.72	1.39	17.40	34.70	19.00
Netherlands	79.20	2.16	88.58	7.39	3.77	2.93	7.30	49.40	16.70
Austria	76.20	3.17	102.66	33.43	3.60	3.16	7.30	40.70	17.50
Poland	72.20	1.21	87.42	11.28	2.66	1.89	4.80	45.70	18.90
Portugal	75.40	1.36	118.90	30.32	2.20	1.64	11.80	33.50	21.60
Romania	69.90	0.50	46.84	23.88	1.66	1.20	16.40	24.60	32.50
Slovenia	75.40	1.95	94.35	21.15	3.23	2.41	4.20	42.70	16.20
Slovakia	72.40	0.84	59.16	11.90	2.90	2.04	8.60	37.70	16.30
Finland	76.30	2.75	81.41	41.16	5.98	4.69	8.30	44.20	16.50
Sweden	82.40	3.32	75.28	54.65	4.62	3.16	7.50	51.80	18.00
United Kingdom	78.70	1.70	61.59	11.02	2.66	2.03	10.70	48.80	23.10

Source: Eurostat (Europe 2020 headline indicators [21]).

**Table 5:** Normalized Europe 2020 headline indicators values in 2016.

	C_1_	C_2_	C_3_	C_4_	C_5_	C_6_	C_7_	C_8_	C_9_
Belgium	0.1796	0.2697	0.8163	0.0712	0.7591	0.7693	0.8403	0.2034	0.8403
Bulgaria	0.1796	0.0824	0.8689	0.1532	0.8631	0.9034	0.7495	0.1508	0.6913
Czech Republic	0.2035	0.1798	0.8520	0.1219	0.7898	0.8316	0.8802	0.1463	0.8984
Denmark	0.2016	0.3306	0.8348	0.2600	0.8294	0.8164	0.8639	0.2075	0.8716
Germany	0.2085	0.3146	0.8338	0.1216	0.7993	0.8110	0.8131	0.1481	0.8495
Estonia	0.2032	0.1338	0.8902	0.2343	0.7516	0.8454	0.8022	0.2026	0.8136
Ireland	0.1894	0.1252	0.7460	0.0756	0.8287	0.8242	0.8911	0.2436	0.8136
Greece	0.1491	0.1059	0.7989	0.1257	0.8823	0.8887	0.8875	0.1905	0.7280
Spain	0.1695	0.1273	0.7389	0.1423	0.8577	0.8732	0.6551	0.1789	0.7868
France	0.1857	0.2376	0.8086	0.1281	0.8003	0.8395	0.8403	0.1950	0.8609
Croatia	0.1629	0.0920	0.8294	0.2309	0.8936	0.8865	0.9492	0.1307	0.7868
Italy	0.1634	0.1466	0.8077	0.1422	0.8648	0.8631	0.7495	0.1169	0.7708
Cyprus	0.1823	0.0556	0.6616	0.0805	0.8413	0.8506	0.8621	0.2382	0.7884
Latvia	0.1942	0.0471	0.9024	0.3033	0.8793	0.8610	0.8185	0.1910	0.7823
Lithuania	0.1995	0.0899	0.9042	0.2092	0.8841	0.8735	0.9129	0.2619	0.7700
Luxembourg	0.1876	0.1391	0.8029	0.0444	0.6009	0.4979	0.9002	0.2436	0.8487
Hungary	0.1897	0.1273	0.8533	0.1169	0.8662	0.8701	0.7749	0.1472	0.7991
Malta	0.1886	0.0610	0.8121	0.0507	0.9127	0.9078	0.6515	0.1428	0.8449
Netherlands	0.2046	0.2140	0.7948	0.0476	0.7886	0.7900	0.8548	0.2039	0.8724
Austria	0.1985	0.3339	0.7691	0.2725	0.7968	0.7685	0.8748	0.1789	0.8625
Poland	0.1839	0.1027	0.8105	0.0920	0.8616	0.8744	0.9056	0.1990	0.8327
Portugal	0.1873	0.1370	0.7416	0.2521	0.8834	0.8878	0.7459	0.1544	0.8082
Romania	0.1759	0.0514	0.8963	0.2044	0.9141	0.9194	0.6642	0.1142	0.7036
Slovenia	0.1860	0.2151	0.7878	0.1739	0.8244	0.8307	0.9111	0.1972	0.8594
Slovakia	0.1852	0.0845	0.8707	0.0982	0.8430	0.8627	0.8657	0.1405	0.8617
Finland	0.1947	0.2911	0.8137	0.3186	0.6725	0.6713	0.8566	0.2057	0.8732
Sweden	0.2154	0.3478	0.8275	0.4359	0.7446	0.7670	0.8657	0.2275	0.8602
United Kingdom	0.2056	0.1776	0.8571	0.0734	0.8483	0.8535	0.7967	0.2146	0.8304

Source: Author’s calculations.

**Table 6:** Calculation of interval entropy for data source R_1_.

$R_{1}^{*}$	0.25	0.30	0.85	0.9
$d_{i}$	2	3	3	2
$p_{i}$	0.2	0.3	0.3	0.2

Source: Author’s calculations.

**Table 7:** Calculation of interval entropy for data source R_1_.

$\left[a_{i-1},\;a_{i}\right)$	[0.240, 0.305)	[0.305,0.370)	[0.370, 0.435)	[0.435, 0.500)	[0.500, 0.565)
$d_{i}$	3	2	0	0	0
$p_{i}$	0.3	0.2	0	0	0
$\left[a_{i-1},\;a_{i}\right)$	[0.565, 0.630)	[0.630, 0.695)	[0.695, 0.760)	[0.760, 0.825)	[0.825, 0.890)
$d_{i}$	0	0	0	0	5
$p_{i}$	0	0	0	0	0.5

Source: Author’s calculations.

**Table 8:** Calculation of interval entropy for data sources R_3_ and R_4_ and k = 2.

**R_3_**	[0.1, 0.55)	[0.55, 1.0)
**d_i_**	5	5
**p_i_**	0.5	0.5
**R_4_**	[0.1, 0.55)	[0.55, 1.0)
**d_i_**	4	6
**p_i_**	0.4	0.6

Source: Author’s calculations.

**Table 9:** Calculation of interval entropy for data sources R_3_ and R_4_ and k = 5.

**R_3_, R_4_**	[0.1, 0.28)	[0.28, 0.46)	[0.46, 0.64)	[0.64, 0.82)	[0.82, 1.0)
**d_i_**	2	2	2	2	2
**p_i_**	0.2	0.2	0.2	0.2	0.2

Source: Author’s calculations.

**Table 10:** Entropy values for indicators C_1_–C_9_ in 2016.

	C_1_	C_2_	C_3_	C_4_	C_5_	C_6_	C_7_	C_8_	C_9_
E(R)	0.9851	0.9851	1.0000	0.9851	1.0000	0.9851	0.9554	0.9703	0.9703
E_2_	0.8631	0.9059	0.6769	0.7496	0.5917	0.3712	0.8631	0.9852	0.9059
E_4_	0.9147	0.9509	0.7995	0.7990	0.7763	0.5492	0.9147	0.9225	0.9337
E_7_	0.8861	0.9343	0.8166	0.8214	0.7820	0.6357	0.8853	0.8687	0.8977
E_8_	0.8787	0.9220	0.8420	0.8505	0.8255	0.6158	0.8607	0.8935	0.8401
E_14_	0.8877	0.8966	0.7944	0.8502	0.7948	0.6924	0.8662	0.8582	0.8653
E_16_	0.8593	0.8713	0.7864	0.8628	0.8136	0.6815	0.8086	0.8517	0.8356
E_2,4,7,14_	0.8879	0.9219	0.7719	0.8050	0.7362	0.5621	0.8823	0.9086	0.9007
E_2,4,8,16_	0.8790	0.9125	0.7762	0.8155	0.7518	0.5544	0.8618	0.9132	0.8788

Source: Author’s calculations.

**Table 11:** Entropy values for indicators C_1_–C_9_ in 2017.

	C_1_	C_2_	C_3_	C_4_	C_5_	C_6_	C_7_	C_8_	C_9_
E(R)	0.9703	0.9851	0.9703	1.0000	0.9851	1.0000	0.9703	0.9703	0.9851
E_2_	0.7496	0.8631	0.6769	0.7496	0.4912	0.3712	0.8631	0.9852	0.9059
E_4_	0.8534	0.9020	0.8086	0.7990	0.7157	0.5089	0.9015	0.9225	0.9392
E_7_	0.8822	0.9089	0.8773	0.8564	0.7820	0.6424	0.9265	0.8630	0.8793
E_8_	0.8760	0.8955	0.8564	0.8459	0.7954	0.6012	0.8848	0.9341	0.8940
E_14_	0.8628	0.8662	0.8815	0.8723	0.8145	0.6585	0.8628	0.8653	0.8850
E_16_	0.8347	0.8245	0.8534	0.8414	0.8034	0.6605	0.8780	0.8950	0.8578
E_2,4,7,14_	0.8370	0.8851	0.8111	0.8193	0.7009	0.5453	0.8885	0.9090	0.9023
E_2,4,8,16_	0.8284	0.8713	0.7988	0.8090	0.7014	0.5355	0.8819	0.9342	0.8992

Source: Author’s calculations.

**Table 12:** Entropy values for indicators C_1_–C_9_ in 2018.

	C_1_	C_2_	C_3_	C_4_	C_5_	C_6_	C_7_	C_8_	C_9_
E(R)	0.9554	0.9851	1.0000	1.0000	0.9851	0.9851	0.9554	0.9498	1.0000
E_2_	0.7496	0.9403	0.6769	0.6769	0.5917	0.3712	0.9059	0.9666	0.9666
E_4_	0.8654	0.9621	0.7995	0.767	0.7608	0.5089	0.9454	0.9020	0.9629
E_7_	0.8947	0.9393	0.8539	0.8521	0.7617	0.6357	0.9406	0.9808	0.9302
E_8_	0.8897	0.8743	0.8445	0.8294	0.7646	0.5996	0.8935	0.8790	0.9356
E_14_	0.8769	0.8769	0.8511	0.8591	0.7834	0.6549	0.9366	0.8896	0.9003
E_16_	0.8748	0.8737	0.8534	0.8338	0.7596	0.6704	0.8482	0.8097	0.8458
E_2,4,7,14_	0.8466	0.9297	0.7954	0.7888	0.7244	0.5427	0.9321	0.9347	0.9400
E_2,4,8,16_	0.8449	0.9126	0.7936	0.7768	0.7192	0.5375	0.8983	0.8893	0.9277

Source: Author’s calculations.

**Table 13:** Indicator weight values and the goal function (12) values for 2016–2018.

**2016**									
Wx_4_	Wx_3_	Wx_5_	Wx_6_	Wy_2_	Wy_8_	Wy_9_	Wy_1_	Wy_7_	$F\left(W_{X}^{0},W_{Y}^{0}\right)$
0.8173	0.1816	0.0011	0	0.9290	0.0182	0.0182	0.0173	0.0173	204.109514
**2017**									
Wx_4_	Wx_3_	Wx_5_	Wx_6_	Wy_8_	Wy_9_	Wy_7_	Wy_2_	Wy_1_	$F\left(W_{X}^{0},W_{Y}^{0}\right)$
0.6719	0.3281	0	0	0.4019	0.1572	0.1572	0.1419	0.1419	230.062190
**2018**									
Wx_3_	Wx_4_	Wx_5_	Wx_6_	Wy_9_	Wy_2_	Wy_7_	Wy_8_	Wy_1_	$F\left(W_{X}^{0},W_{Y}^{0}\right)$
0.5745	0.4135	0.0099	0.0021	0.3482	0.3086	0.2385	0.1042	0.0005	220.053163

Source: Author’s calculations.

**Table 14:** α values, α-cuts, and final ranks of European countries for 2016.

α	A_α_	Country	No	Rank
0.3616	27	Sweden	27	1
0.3477	20,27	Austria	20	2
0.3452	4,20,27	Denmark	4	3
0.3082	4,20,26,27	Finland	26	4
0.2524	4,10,20,26,27	France	10	5
0.2517	4,5,10,20,26,27	Germany	5	6
0.2380	4,5,10,20,24,26,27	Slovenia	24	7
0.2072	1,4,5,10,20,24,26,27	Belgium	1	8
0.2048	1,3,4,5,10,20,24,26,27	Czech Republic	3	9
0.2014	1,3,4,5,10,20,24,26,27,28	UK	28	10
0.1841	1,3,4,5,10,19,20,24,26,27,28	Netherlands	19	11
0.1681	1,3,4,5,10,12,19,20,24,26,27,28	Italy	12	12
0.1679	1,3,4,5,10,12,16,19,20,24,26,27,28	Luxembourg	16	13
0.1609	1,3,4,5,10,12,16,19,20,22,24,26,27,28	Portugal	22	14
0.1601	1,3,4,5,6,10,12,16,19,20,22,24,26,27,28	Estonia	6	15
0.1542	1,3,4,5,6,7,10,12,16,19,20,22,24,26,27,28	Ireland	7	16
0.1522	1,3,4,5,6,7,10,12,16,17,19,20,22,24,26,27,28	Hungary	17	17
0.1501	1,3,4,5,6,7,9,10,12,16,17,19,20,22,24,26,27,28	Spain	9	18
0.1330	1,3,4,5,6,7,8,9,10,12,16,17,19,20,22,24,26,27,28	Greece	8	19
0.1330	1,3,4,5,6,7,8,9,10,12,16,17,19,20,21,22,24,26,27,28	Poland	21	20
0.1215	1,3,4,5,6,7,8,9,10,12,15,16,17,19,20,21,22,24,26,27,28	Lithuania	15	21
0.1214	1,3,4,5,6,7,8,9,10,11,12,15,16,17,19,20,21,22,24,26,27,28	Croatia	11	22
0.1150	1,3,4,5,6,7,8,9,10,11,12,15,16,17,19,20,21,22,24,25,26,27,28	Slovakia	25	23
0.1079	1,2,3,4,5,6,7,8,9,10,11,12,15,16,17,19,20,21,22,24,25,26,27,28	Bulgaria	2	24
0.0892	1,2,3,4,5,6,7,8,9,10,11,12,15,16,17,18,19,20,21,22,24,25,26,27,28	Malta	18	25
0.0884	1,2,3,4,5,6,7,8,9,10,11, 12,13,15,16,17,18,19,20,21,22,24,25,26,27,28	Cyprus	13	26
0.0790	1,2,3,4,5,6,7,8,9,10,11,12,13,14,15,16,17,18,19,20,21,22,24,25,26,27,28	Latvia	14	27
0.0771	1,2,3,4,5,6,7,8,9,10,11,12,13,14,15,16,17,18,19,20,21,22,23,24,25,26,27,28	Romania	23	28

Source: Author’s calculations.

**Table 15:** α values, α-cuts, and final ranks of European countries for 2017.

α	A_α_	Country	No	Rank
0.4409	27	Sweden	27	1
0.4262	4,27	Denmark	4	2
0.4178	4,26,27	Finland	26	3
0.4156	4,20,26,27	Austria	20	4
0.4055	4,15,20,26,27	Lithuania	15	5
0.3873	4,6,15,20,26,27	Estonia	6	6
0.3739	4,6,15,20,24,26,27	Slovenia	24	7
0.3666	4,6,14,15,20,24,26,27	Latvia	14	8
0.3604	3,4,6,14,15,20,24,26,27	Czech Republic	3	9
0.3593	3,4,6,11,14,15,20,24,26,27	Croatia	11	10
0.3585	3,4,5,6,11,14,15,20,24,26,27	Germany	5	11
0.3545	3,4,5,6,11,14,15,20,22,24,26,27	Portugal	22	12
0.3519	3,4,5,6,10,11,14,15,20,22,24,26,27	France	10	13
0.3518	3,4,5,6,8,10,11,14,15,20,22,24,26,27	Greece	8	14
0.3496	3,4,5,6,8,10,11,14,15,17,20,22,24,26,27	Hungary	17	15
0.3470	3,4,5,6,8,10,11,14,15,17,20,22,24,25,26,27	Slovakia	25	16
0.3352	3,4,5,6,8,10,11,14,15,17,20,22,24,25,26,27,28	UK	28	17
0.3350	3,4,5,6,8,9,10,11,14,15,17,20,22,24,25,26,27,28	Spain	9	18
0.3284	3,4,5,6,8,9,10,11,12,14,15,17,20,22,24,25,26,27,28	Italy	12	19
0.3240	2,3,4,5,6,8,9,10,11,12,14,15,17,20,22,24,25,26,27,28	Bulgaria	2	20
0.3238	2,3,4,5,6,8,9,10,11,12,14,15,17,20,21,22,24,25,26,27,28	Poland	21	21
0.3177	1,2,3,4,5,6,8,9,10,11,12,14,15,17,20,21,22,24,25,26,27,28	Belgium	1	22
0.3034	1,2,3,4,5,6,7,8,9,10,11,12,14,15,17,20,21,22,24,25,26,27,28	Ireland	7	23
0.2999	1,2,3,4,5,6,7,8,9,10,11,12,14,15,17,18,20,21,22,24,25,26,27,28	Malta	18	24
0.2975	1,2,3,4,5,6,7,8,9,10,11,12,14,15,17,18,19,20,21,22,24,25,26,27,28	Netherlands	19	25
0.2965	1,2,3,4,5,6,7,8,9,10,11,12,14,15,16,17,18,19,20,21,22,24,25,26,27,28	Luxembourg	16	26
0.2963	1,2,3,4,5,6,7,8,9,10,11,12,14,15,16,17,18,19,20,21,22,23,24,25,26,27,28	Romania	23	27
0.2724	1,2,3,4,5,6,7,8,9,10,11,12,13,14,15,16,17,18,19,20,21,22,23,24,25,26,27,28	Cyprus	13	28

Source: Author’s calculations.

**Table 16:** α values, α-cuts, and final ranks of European countries for 2018.

α	A_α_	Country	No	Rank
0.6289	27	Sweden	27	1
0.6082	26,27	Finland	26	2
0.6081	4,26,27	Denmark	4	3
0.5618	4,20,26,27	Austria	20	4
0.5506	3,4,20,26,27	Czech Republic	3	5
0.5479	3,4,5,20,26,27	Germany	5	6
0.5455	3,4,5,11,20,26,27	Croatia	11	7
0.5440	3,4,5,11,20,25,26,27	Slovakia	25	8
0.5420	3,4,5,11,20,25,26,27,28	UK	28	9
0.5391	3,4,5,11,15,20,25,26,27,28	Lithuania	15	10
0.5368	3,4,5,11,15,17,20,25,26,27,28	Hungary	17	11
0.5328	3,4,5,11,15,17,20,24,25,26,27,28	Slovenia	24	12
0.5322	3,4,5,10,11,15,17,20,24,25,26,27,28	France	10	13
0.5302	3,4,5,6,10,11,15,17,20,24,25,26,27,28	Estonia	6	14
0.5286	3,4,5,6,10,11,15,17,20,22,24,25,26,27,28	Portugal	22	15
0.5278	3,4,5,6,8,10,11,15,17,20,22,24,25,26,27,28	Greece	8	16
0.5099	3,4,5,6,8,10,11,15,17,20,21,22,24,25,26,27,28	Poland	21	17
0.5090	1,3,4,5,6,8,10,11,15,17,20,21,22,24,25,26,27,28	Belgium	1	18
0.5059	1,3,4,5,6,8,10,11,14,15,17,20,21,22,24,25,26,27,28	Latvia	14	19
0.4982	1,3,4,5,6,8,10,11,12,14,15,17,20,21,22,24,25,26,27,28	Italy	12	20
0.4951	1,3,4,5,6,8,10,11,12,14,15,17,19,20,21,22,24,25,26,27,28	Netherlands	19	21
0.4908	1,3,4,5,6,8,10,11,12,14,15,16,17,19,20,21,22,24,25,26,27,28	Luxembourg	16	22
0.4882	1,3,4,5,6,8,9,10,11,12,14,15,16,17,19,20,21,22,24,25,26,27,28	Spain	9	23
0.4865	1,3,4,5,6,8,9,10,11,12,14,15,16,17,18,19,20,21,22,24,25,26,27,28	Malta	18	24
0.4756	1,3,4,5,6,7,8,9,10,11,12,14,15,16,17,18,19,20,21,22,24,25,26,27,28	Ireland	7	25
0.4729	1,2,3,4,5,6,7,8,9,10,11,12,14,15,16,17,18,19,20,21,22,24,25,26,27,28	Bulgaria	2	26
0.4448	1,2,3,4,5,6,7,8,9,10,11,12,14,15,16,17,18,19,20,21,22,23,24,25,26,27,28	Romania	23	27
0.4336	1,2,3,4,5,6,7,8,9,10,11,12,13,14,15,16,17,18,19,20,21,22,23,24,25,26,27,28	Cyprus	13	28

Source: Author’s calculations.

**Table 17:** Comparison of the clustering solution calculated in the current research with the categorization results by Fedajev et al. [4].

	Rank 2016	Rank 2017	Rank 2018	Three Clusters k-Means	Rank Fedajev et al.	Category Fedajev et al.	Four Clusters k-Means
Belgium	8	22	18	3	20	Periphery	3
Bulgaria	24	20	26	3	24	Periphery	3
Czech Republic	9	9	5	1	9	Core	1
Denmark	3	2	3	1	2	Core	1
Germany	6	11	6	1	11	Semi-Periphery	1
Estonia	15	6	14	2	10	Semi-Periphery	4
Ireland	16	23	25	3	19	Semi-Periphery	3
Greece	19	14	16	2	16	Semi-Periphery	4
Spain	18	18	23	3	25	Periphery	3
France	5	13	13	1	8	Core	1
Croatia	22	10	7	2	6	Core	2
Italy	12	19	20	3	22	Periphery	3
Cyprus	26	28	28	3	26	Periphery	3
Latvia	27	8	19	2	12	Semi-Periphery	4
Lithuania	21	5	10	2	4	Core	4
Luxembourg	13	26	22	3	28	Periphery	3
Hungary	17	15	11	2	18	Semi-Periphery	2
Malta	25	24	24	3	27	Periphery	3
Netherlands	11	25	21	3	22	Periphery	3
Austria	2	4	4	1	3	Core	1
Poland	20	21	17	3	13	Semi-Periphery	3
Portugal	14	12	15	2	15	Semi-Periphery	4
Romania	28	27	27	3	20	Periphery	3
Slovenia	7	7	12	1	5	Core	1
Slovakia	23	16	8	2	17	Semi-Periphery	2
Finland	4	3	2	1	7	Core	1
Sweden	1	1	1	1	1	Core	1
UK	10	17	9	2	14	Semi-Periphery	2

Source: Author’s calculations.

## Data Availability

The authors have used publicly archived Eurostat dataset named Europe 2020 Headline Indicators. The dataset is available at https://ec.europa.eu/eurostat/web/europe-2020-indicators/europe-2020-strategy/main-tables (accessed on 10 January 2021).
